# Defining a Brief Intervention for the Promotion of Psychological Well-being among Unemployed Individuals through Expert Consensus

**DOI:** 10.3389/fpsyt.2018.00013

**Published:** 2018-02-07

**Authors:** Osvaldo Santos, Elisa Lopes, Ana Virgolino, Miodraga Stefanovska-Petkovska, Alexandra Dinis, Sara Ambrósio, Maria João Heitor

**Affiliations:** ^1^Faculdade de Medicina, Universidade de Lisboa, Lisbon, Portugal; ^2^Instituto de Medicina Preventiva e Saúde Pública, Faculdade de Medicina, Universidade de Lisboa, Lisbon, Portugal; ^3^Instituto de Saúde Ambiental, Faculdade de Medicina, Universidade de Lisboa, Lisbon, Portugal; ^4^Hospital Beatriz Ângelo, Loures, Portugal

**Keywords:** unemployment, well-being, mental health promotion, Delphi technique, consensus development

## Abstract

**Background:**

Epidemiologic evidence highlights the harmful consequences of unemployment on health and well-being. This emphasizes the need to design low-cost interventions to prevent the adverse mental health effects of unemployment. The main aim of this study was to create expert-consensus regarding development and implementation of a brief, sustainable, and effective intervention program for promoting mental health among unemployed.

**Methods:**

The Delphi technique entailed a selected panel of 75 experts from various relevant professional backgrounds. Panel members were asked to state their level of agreement (5-point Likert scale) regarding (a) required characteristics for an effective mental health intervention for unemployed people and (b) key variables for assessing the effectiveness of that intervention. Consensus was obtained throughout two rounds of data collection through e-mail contact, with structured questionnaires. Items of the questionnaire were based on literature reviews about community-based interventions for unemployed individuals.

**Results:**

Overall, 46 experts collaborated with the Delphi process (final participation rate: 61.3%). Based on a review of the literature, 185 items were identified and grouped into two broad categories (set of characteristics of the intervention and set of variables for effectiveness assessment), aggregating a total of 11 dimensions. The two Delphi rounds resulted in the selection of 35 characteristic items for the intervention program and 54 variables for its effectiveness assessment. Brief group interventions were considered to be effective and sustainable for mental health promotion in unemployment conditions if targeting mental health literacy, training interpersonal skills, and job-search skills.

**Conclusion:**

As agreed by the panel of experts, a brief, sustainable and effective intervention can be developed and implemented by accounting for unemployed capacity-building for mental health self-care and adequate job-searching attitudes and skills. These results should be further implemented in community and multisector-based standardized interventions, targeting mental health among unemployed people, ensuring adequate conditions for its effectiveness assessment.

## Introduction

Job loss represents an involuntary and disruptive event with multiple and extended impact on individuals’ life. The recent European Union economic crises have directed the attention of scientists and policy makers to the effects of job loss and unemployment. In Portugal, the deterioration of individuals’ socioeconomic conditions (1) and the increase of unemployment rates are among the most striking reflections of this economic turmoil. Despite witnessing significant post-crises improvement in the labor market sector, in December 2016, the unemployment rate in Portugal was 10.9% (4.1 percentage-points higher than the OECD average), with a 27.7% rate among young adults (less than 25 years old) (2). Particularly concerning was the high proportion of unemployed for 1 year or more (55.0% in Portugal and 33.0% OECD average). Portugal has the highest prevalence of mental health illness (22.9% according to the World Mental Health Survey) (3) while current life satisfaction of Portuguese people remains comparable as in the time of the economic crises—the bottom third of the OECD country ranking (4). These socioeconomic and labor market trends raise the ongoing debate about the relationship between unemployment and individual well-being.

Different epidemiologic studies from Europe and United States have shown that a low socioeconomic status represents a risk factor for illness and mortality (5). More specifically, increases in the unemployment rate, particularly long-term unemployment (5, 6), are related to increased risk of heart disease, stroke mortality, mental illness, and suicide (7–10). In addition, the combination of economic crises with decreased access to health care (due to austerity measures) has been found to have negative effects on mental health, with a robust evidence regarding depression symptoms and suicide (11, 12), somatic and mood disorders (e.g., depression, anxiety), as well as alcohol and drug abuse (11). In the long run, socioeconomic status is characterized with inter-generational impact by determining children’s level of education, thus influencing their future probability of employment and income stability. Finally, socioeconomic conditions are also reflected on the individual-level interpersonal and professional achievements (11).

The World Health Organization argues that the negative implication of unemployment on individual mental health can be prevented (12). Recent research highlights the need to design interventions for preventing health deterioration and monitoring health specific risks among vulnerable (namely unemployed) groups (13). However, the majority of programs that were developed to minimize adverse mental health effects of unemployment were designed in the pre-crises period and mainly target the promotion of re-employment skills. Also, contexts of a depressed economy tend to limit the efforts for mental health promotion due to lack of resources and also due to lack of adequate scientific-evidence about mental health promotion/prevention programs. Although Portugal has one of the highest prevalence of lifetime mental disorders, very few studies investigated the unemployment-mental-health association, within the context of the Portuguese economic downturn. More specifically, the available studies focus on analyzing the impact of unemployment on suicide rates (14), family life, children and adolescents’ well-being (15, 16), as well as on most vulnerable ethnic groups (e.g., Roma) (17). Further research on the effect of unemployment on mental health of Portuguese citizens is needed with special emphasis on raising awareness about the role of mental health promotion and its effectiveness when based on large-scale public health policy strategies (15).

The main aim of this study was to create expert consensus regarding how to develop and implement a brief and sustainable intervention program for promoting mental health among unemployed individuals, which is able to counterbalance the potential negative mental health effects of unemployment. This was done within the context of the Healthy Employment project (reference: 222SM2), a funded project by Public Health Initiatives Programme (PT06), through the EEA Grants Financial Mechanism 2009–2014 that aims to develop and evaluate the effectiveness of an intervention for mental health promotion among unemployed persons: this funding program targeted mental health and mental health promotion actions as one of its priority areas. This is especially relevant for Portugal when considering that mental health-care services are not easily accessible for the majority of the population due to lack of specialized public health professionals, which affects the coverage of the needs of the most economically vulnerable population.

## Materials and Methods

The Delphi method is a consensus-building method designed to enhance effective decision-making in health, educational, and/or social care (just to name some of the possible areas of application). It is defined as a method for structuring a group communication process focused on creating consensus about complex problems.

It consists of a group facilitation technique based on a multistage process designed to transform expert opinion into group consensus. An expert is defined as one who is considered to be knowledgeable about the subject under consideration and capable of representing the views of his or her peers. Panel members participate by responding anonymously to a questionnaire/form that is adapted throughout different rounds of answers. The initial version of the questionnaire or form is usually developed on basis of literature review and/or other qualitative data collection (e.g., focus groups, in-depth interviews). The anonymity of responses minimizes peer pressure and enhances the flow and exchange of ideas.

In comparison to other methods for obtaining expert opinion and consensus, such as nominal group process or expert round tables (18), the Delphi method has advantages, such as: (1) possibility to include asynchronous inputs from experts of different profiles, backgrounds, and geographical location; (2) flexible time to respond (while respecting pre-defined deadlines); (3) expert’s answers are anonymous to the rest of the panel (only accessible to researchers), avoiding peer-to-peer pressure and specific opinion-makers’ influence (e.g., from highly respected experts); (4) iterative nature of the method, with structured feedback from researchers; and (5) possibility to explore complex and multidimensional topics with limited scientific evidence or clarity (19–23).

### Expert Panel Selection and Procedure

This project received approval from the Ethics Committee of the Centro Académico de Medicina de Lisboa and from the Comissão Nacional de Proteção de Dados. Experts were recruited using the snow ball sampling method. Identification of experts was made by recommendation from the research participants, relevant authorities, and stakeholders in the area. During the selection process, special considerations were made to the following (non-cumulative) criteria:

– Knowledge and professional experience in the area of mental health, employment/temporary work, public mental health programs, and prevention of psychiatric disorders;
– Managerial functions in organizations dealing with mental health and/or employment;
– Scientific or organizational work in the fields of mental health, unemployment, and/or economic crises.

Deliberate effort was made to provide diversity in terms of years of professional experience and geographic distribution. The selection process ensured the representation of relevant institutions working on mental health or labor.

Formal invitations for participation in the panel were sent to the identified experts *via* email. The invitations consisted of (1) information on the background and objectives of the study, anonymous and voluntary participation and (2) short description and instructions regarding the Delphi method. An informed consent form was provided to all experts prior to proceeding further in the study. Only those who indicated to be willing to participate in the study integrated the Delphi panel.

### Data Collection Process

This Delphi study involved two rounds of data collection conducted between August and September 2015. In both rounds, the answer form was sent (always by the same researcher) simultaneously to all experts of the panel (all of them in bcc field, to ensure anonymity). Only experts who participated in the first round were invited to participate in the second round. This decision was taken due to the high participation rate of the first round.

In the first round, experts who did not respond to the first invitation email were sent a second and a third reminder within an interval of 1–2 weeks. Those who did not respond to the three calls were excluded from the sample and considered as non-responders in the first round. In the second round, a reminder was emailed to all participants who did not answer the first second-round call.

### Formulation of Items and Answer Format

Both contents (item generation) and structure of the questionnaire were based on a review of the literature, conducted on the PubMed, SciELO, and EBSCO databases by two researchers (one psychologist and one sociologist), with the supervision of two other researchers (psychologists). Special consideration was given to unemployment intervention programs. The identified articles were summarized in a synoptic table and categorized by the following descriptors: (a) type of article, (b) target group, (c) characteristics of participants (exclusion and inclusion criteria), (d) theoretical models of intervention, (e) psychological functioning areas, (f) geographical region of intervention, (g) recruitment method, (h) settings, (i) format of the intervention, (j) frequency of sessions, (k) number of sessions, (l) total duration of the intervention, (m) areas of intervention, (n) assessment tools, (o) training materials, (p) number of assessments, and (q) main results. Based on this review, a total of 185 items were identified as relevant to present to the Delphi panel, distributed in two broad sections (categories), aggregating 11 sub-sections (dimensions).

The questionnaire structure (wording and answer format) was tailored to the multidisciplinary profile of the panel. Two main types of response formats were used in the questionnaire. Answers regarding paradigms, models, contents, and skills to promote by the intervention were collected through a 5-point Likert scale (where “1 = totally disagree” and “5 = totally agree”). Experts were asked to rate each item according to this scale, with no limitation of the number of items they could endorse total agreement. They were instructed to use the option “*no opinion*” in cases when they believed that the item did not relate to their area of expertise or when not having sufficient relevant expertise. Single-choice answer format was used for acquiring experts’ opinions regarding different operational aspects of the intervention (e.g., number of sessions, frequency and duration of sessions, number of participants per intervention, location for implementation). Items were organized in two main sections/categories: intervention development: structure and competences to promote, and indicators/variables required for effectiveness evaluation. Each category was divided in different subsections/dimensions. At the end of each subsection/dimension, experts were invited to make comments, propose new items, and/or reformulate the items for which they attributed a low score (1–3).

### First Round

For the first round, experts received the questionnaire and instructions by email. All participants were asked to provide their general sociodemographic and professional-related information. They were encouraged to provide comments on each indicator using open text boxes, namely proposing changes of wording and/or additional important indicators. Those proposed or amended items were submitted for consensus in the second round.

### Second Round

Only experts who have completed the first-round questionnaire were sent the second-round questionnaire, also by email. This second-round questionnaire included the new/reformulated items and a brief report with the results from the first round, listing the items, which obtained consensus, as well as the percentage of agreement with each of the items that passed to the second round (because of lacked consensus). Comments that were given by experts within the first round to the items that did not receive consensus in the first round were also included. The possibility to include additional items, to comment, or to change the items-formulation was also available during this second round.

### Consensus Criteria

Although there are no definite consensus criteria regarding the Delphi studies, in the majority of available research, the predefined consensus level ranges from 60 to 80% (24). For this study, consensus was based on the following cumulative criteria: minimal response rate (per round) equal or higher than 85%, average score equal or higher than 3.5 (on a 5-point scale), coefficient of variation (i.e., ratio of the SD to the mean) lower than 0.35 and 80% or more of experts attributing an agreement of 4 or 5, in the 5-points scale.

### Data Analyses

Data analyses were done in Excel 15.0 and SPSS 23.0. Descriptive analyses were conducted for each Delphi round, with computation of percentages, mean values, and coefficients of variation (SD by mean). Consensus flag variables were created based on previously explained criteria. Thematic content analysis was also performed for the construction of new items (from the first to the second round). At the moment of launching the second round, panel members received the list of items that were approved, the list of items that were deleted and the new/reformulated items that required their appreciation. They were informed about the consensus criteria but did not get the percentage of agreement per item.

## Results

This section presents the results from the Delphi panel. Results are reported using textual and tabular presentations, outlining means and variance for each indicator, as well as percentage of responses scoring higher than 3 on the 5-points response scale (19, 23).

From a total of 75 identified and invited experts, 51 accepted the invitation (initial participation rate: 68.0%). The majority of the 24 participants who did not respond to the invitation were psychologists or psychiatrists, mainly from the Metropolitan Area of Lisbon. From the number of experts that agreed to be included in the panel, 46 answered the questionnaire (answer rate for the first round: 90.0%). The answer rate of the second round was 100% (*n* = 46; final participation rate, considering all initially invited experts: 61.3%).

The sociodemographic and professional characteristics of the experts are presented in Table 1. The average age of experts was 48.17 ± 12.48 years. Most represented professional categories were psychologists (34.8%), psychiatrists (17.4%), and sociologists (15.2%). A relevant number of experts had a doctorate degree (47.8%) and worked in their field of intervention for 18.74 ± 9.94 years. Regarding the geographical distribution, at the time of the Delphi study, 60.9% worked in the Metropolitan Area of Lisbon.

**Table 1:** Sociodemographic profile of Delphi panel experts (*n* = 46).

		*n*	%
Professional area	Family medicine	3	6.5
	Psychiatry	8	17.4
	Public health (medicine)	6	13.0
	Psychology	16	34.8
	Social workers	3	6.5
	Sociology	7	15.2
	Economic sciences	1	2.2
	Social politics	2	4.3

Academic degree	Bachelor	12	26.1
	Master degree	12	26.1
	Ph.D.	22	47.8

Years of professional experience	Less than 5 years	2	4.3
	5–9 years	6	13.0
	10–19 years	14	30.4
	20 years or more	24	52.2

Age	Less than 35 years old	9	19.6
	35–44 years old	11	23.9
	45–54 years old	8	17.4
	54–64 years old	14	30.4
	65+ years old	4	8.7

Region	North	6	13.0
	Center	5	10.9
	Lisbon Metropolitan Area	28	60.9
	Alentejo	4	8.7
	Algarve	3	6.5

Table 2 summarizes the number of items assessed by the experts in each of the two rounds. Based on the conducted literature review, a total of 145 items regarding unemployment were administered to experts in the first round, aggregated into 11 categories. The second round contained 40 new or reformulated items. The items that were not retained for the final intervention are presented as supplementary data.

**Table 2:** Number of items submitted to experts in rounds 1 and 2.

Dimensions	Round 1 items	Round 2 items	Total number of submitted items	Total number of approved items (at the end of the Delphi process)
**Category 1—intervention development: structure and competences that should be promoted**				

A—paradigms of intervention	20	2	22	3
B—relational/interpersonal strategies	17	1	18	7
C—format and setting of the intervention	6	–	5	5
D—contents of the intervention	7	6	12	12
E—contents/skills to be developed/promoted	11	8	19	8

**Category 2—relevant indicators/variables, for effectiveness evaluation**				

F—sociodemographic and professional indicators	18	7	25	17
G—Previous employment/unemployment experience	7	4	11	11
H—unemployment financial support	8	3	11	7
I—participants satisfaction with the intervention	13	6	19	11
J—mental health indicators/outcomes	24	2	26	3
K—(re)employment capability	14	1	15	5

Total number of items	145	40	185	89

### Category 1: Intervention Development: Structure and Competences to Promote

The Delphi process resulted in 35 consensual items (aggregated into five dimensions) that can serve as guidelines for operationalizing a mental health promotion intervention (Table 3). Regarding the *Models and paradigms* of the proposed intervention, the experts highlighted the importance of both psycho-education for promotion of mental health literacy (mainly about anxiety, mood disorders, and stigma about mental health, providing didactics about their determinants, maintenance, and treatment factors), cognitive-behavioral models of anxiety and depression (highlighting the interplay between cognitions, namely, automatic thoughts—emotions and behaviors), for promoting the recognition and adaptation to challenges associated with the unemployment situation. Regarding *relational/interpersonal strategies*, the majority of experts pointed the importance of a very pragmatic intervention, focused on hands-on exercises: role plays, problem-solving exercises, sharing of positive/constructive experiences with the situation of unemployment, and exercises for coping effectively with interpersonal conflicts were the most highly appreciated items. Regarding contents of the intervention, consensus was obtained for both mental health promotion skills (e.g., mental health literacy, self-regulation of emotions, effective communication training, awareness of skills, and personal facets to improve) and job-search skills (e.g., job-interviewing training, job-searching habits). Finally, the results indicated that the intervention should focus on the *development of skills* that increase resilience and decrease frustration with the unemployment situation, emotional regulation, and job search skills.

**Table 3:** Consensual items regarding theoretical frameworks, skills/competences to promote, and operational aspects of the intervention (*n* = 46).

Theoretical frameworks and skills/competences to promote in the intervention				

	Mean	Coefficient of variation (σ/μ)	% of agreement^a^	“No opinion” (%)
**A. Models/paradigms of intervention**				
Cognitive-behavioral strategies: adapting to unemployment	4.31	0.17	84.09	4.35
Psychoeducation: mental health literacy	4.39	0.18	81.40	6.52
Motivational interview: principles and strategies	4.36	0.25	81.40	6.52

**B. Relational/interpersonal strategies (exercises, activities)**				
Sharing positive/constructive experiences (exercises)	4.67	0.11	86.96	0.00
Problem solving exercises	4.76	0.09	84.78	0.00
Role play (e.g., for assertive training)	4.59	0.16	84.78	0.00
Management of interpersonal conflicts (exercises)	4.76	0.10	82.61	0.00
Verbal/non-verbal communication (exercises)	4.63	0.12	82.61	0.00
Cognitive-behavioral exercises (e.g., ABC, reality-check)	4.39	0.16	82.61	0.00
Self-regulations of emotions (exercises)	4.58	0.15	80.43	0.00

**C. Operational aspects of the intervention**				
**Number of sessions**			***n***	**%**
4 sessions			1	2.2
6 sessions			6	13.0
8 sessions			15	32.6
10 sessions			9	19.6
>10 sessions			15	32.6

**Size of groups**			***n***	**%**
8 participants			11	23.9
10 participants			21	45.6
12 participants			12	26.1
14 participants			2	4.6
>14 participants			0	0.0

**Frequency of sessions**			***n***	**%**
Daily			8	17.4
Weekly			34	73.9
Every 15 days			3	6.5
Monthly			1	2.2

**Duration of sessions**			***n***	**%**
1 h			4	8.9
2 h			28	60.9

3 h			7	15.2
4 h			6	13.0
6–8 h			1	2.2

**Intervention setting**			***n***	**%**
Employment agencies			26	56.5
Primary health center			3	6.5
Community setting (school, church, …)			16	34.8
Other			1	2.2

**D. Contents of the intervention**				
Recognizing and adapting to challenges imposed by unemployment	4.66	0.18	97.78	2.17
Defining values of life and professional career goals	4.67	0.11	86.96	0.00
Building up job-search habits	4.43	0.17	86.96	0.00
Strategy for contacting potential employers	4.36	0.16	86.67	2.17
Mental health literacy	4.33	0.24	86.36	4.35
Self-awareness of professional interests	4.40	0.16	84.78	0.00
Job-interview training	4.57	0.16	82.61	0.00
Training practical strategies for establishing/enhancing networks	4.44	0.18	82.22	2.17
Self-awareness of personal professional and interpersonal skills/competences	4.52	0.23	82.22	2.17
Assertiveness training	4.52	0.15	80.43	0.00
Stress inoculation	4.64	0.19	80.43	0.00
Personal presentation skills (i.e., personal hygiene, dress code)	4.40	0.18	80.00	2.17

**E. Skills to be developed/promoted**				
Ability to express one’s own ideas (verbal or written)	4.70	0.20	95.56	2.17
Resilience to adversity and frustration tolerance	4.80	0.18	95.56	2.17
Emotional self-regulation	4.66	0.20	95.24	8.70
Job-search skills	4.60	0.27	88.89	2.17
Assertiveness	4.44	0.21	84.78	0.00
Personal presentation skills	4.50	0.24	84.44	2.17
Setback/unsuccessful stress-related inoculation	4.56	0.22	82.61	0.00
Attributional style regarding unemployment situation	4.26	0.26	80.49	10.87

*^a^“4” or “5” on a 5-point response scale*.

Regarding the structure and format of the intervention, experts tend to favor interventions targeted to small groups (up to 10 participants), conducted on more than 10 (weekly) sessions with an approximate duration of 2 h per session. Employment agencies or local community facilities (schools, churches) were the most frequently reported settings for the intervention.

### Category: 2. Indicators/Variables Required for Effectiveness Evaluation

The list of variables/indicators considered as relevant by experts for evaluating the effectiveness of the intervention included 54 items (Table 4). Besides *socioeconomic characteristics and professional indicators*, several aspects related with *professional career and previous unemployment events* were considered as relevant for stratifying and/or adjusting results from the intervention. Additionally, the self-image of a social beneficiary, as well as type/quality of motivation for job search and re-employment were also considered as relevant for evaluating the effectiveness of the intervention. As main outcomes, experts highlighted the importance of (a) participants’ satisfaction with the intervention (as well as assiduity to sessions), and (b) anxiety, satisfaction with family and social life, and general psychosocial functioning, as most relevant indicators of mental health. Finally, some indicators of (re)employment capability and (re)employment potential were proposed to be measured as secondary outcomes.

**Table 4:** Consensual items regarding indicators/variables required for effectiveness evaluation (*n* = 46).

	Mean	Coefficient of variation (σ/μ)	% of agreement	% of “no opinion”
**F. Sociodemographic and professional indicators**				
Age	4.89	0.06	100.00	0.00
Gender	4.85	0.07	100.00	0.00
Level of education	4.87	0.07	100.00	0.00
Family situation (i.e., single, married, widow)	4.47	0.14	95.56	2.17
Professional/academic area(s)	4.59	0.17	93.48	0.00
Number of children	4.48	0.15	93.48	0.00
Household size/composition	4.41	0.16	91.30	0.00
Number of other dependent people (in household)	4.36	0.18	91.11	2.17
Number of unemployed (in household)	4.50	0.22	91.11	2.17
Perceived social support	4.50	0.21	88.89	2.17
Household ability to tackle loans and basic bills	4.41	0.24	88.89	2.17
Overall household financial condition (current)	4.50	0.17	88.64	4.35
Overall household financial condition (before unemployment)	4.41	0.19	86.36	4.35
Area of residence (predominantly urban/predominantly rural)	4.07	0.29	84.09	4.35
If foreign citizen, subjective perception of integration in Portugal	4.21	0.23	84.09	4.35
If foreign citizen, time of residence in Portugal	4.12	0.25	81.40	6.52
Nationality	4.20	0.20	80.00	2.17

**G. Previous employment/unemployment experience**				
Duration of previous job	4.62	0.18	97.83	0.00
Duration of current unemployment situation	4.82	0.17	97.83	0.00
Perception on the determinants of owns unemployment	4.80	0.17	97.83	0.00
Number of previous jobs	4.64	0.19	93.48	0.00
Number of previous unemployment situations	4.67	0.26	93.33	2.17
Type of previous job’s contract	4.51	0.21	89.13	0.00
Professional position in last job	4.42	0.21	89.13	0.00
Overall satisfaction with last job	4.40	0.28	88.89	2.17
Perception of the determinants of unemployment (in general)	4.21	0.31	86.36	4.35
Highest job position ever attained	4.31	0.22	84.78	0.00
Other events of unemployment and/or labor instability in the household family	4.37	0.32	81.82	4.35

**H. Unemployment financial support**				
Self-image as social beneficiary	4.28	0.25	86.36	4.35
Opinions about support received by public employment center	4.36	0.24	86.05	6.52
Knowledge about opportunities to receive social unemployment benefit	4.14	0.28	84.09	4.35
Received unemployment social benefits	4.67	0.11	82.22	2.17
Other social benefits received	4.57	0.13	81.82	4.35
Duration of receiving social benefits	4.51	0.20	81.82	4.35
Motivation for job search/re-employment	4.56	0.16	80.00	2.17

**I. Participants’ satisfaction with the intervention**				
Evaluation of the usefulness of the sessions	4.63	0.11	86.96	0.00
Satisfaction with overall duration of the intervention	4.43	0.15	84.78	0.00
Satisfaction about the location and conditions of the intervention	4.24	0.16	84.78	0.00
Satisfaction with trainers’ skills to motivate participants	4.57	0.15	82.61	0.00
Satisfaction with the frequency/periodicity of sessions	4.46	0.15	82.61	0.00
Self-evaluation of involvement/participation in session	4.67	0.10	82.61	0.00
Evaluating the coherence of/between trainers	4.47	0.25	82.22	2.17
Satisfaction with trainers’ skills to transmit information	4.41	0.16	80.43	0.00
Satisfaction with sessions’ dynamics	4.72	0.11	80.43	0.00
Number of attended sessions	4.50	0.16	80.43	0.00
Evaluating the involvement of trainers	4.57	0.20	80.00	2.17

**J. Mental health indicators/outcomes**				
Anxiety	4.39	0.18	82.61	0.00
Satisfaction with relationships (family/social life)	4.54	0.12	82.61	0.00
Psychosocial functioning	4.45	0.16	82.22	2.17

**K. (Re)employment capability**				
Perceived impact of the intervention for the acquisition of employment	4.50	0.22	84.44	2.17
Perception of self-efficacy for acquiring a new job	4.58	0.20	84.09	4.35
Perception of self-efficacy for attending job interviews	4.67	0.19	81.82	4.35
Satisfaction with new job (type of job, type of contract, …)	4.28	0.23	81.82	4.35
Percentage of participants getting a new job	4.60	0.24	80.00	2.17

## Discussion

Relevant amount of literature identified factors that shape the association between socioeconomic outcomes and periods of exacerbated unemployment rates. The recent European Union crisis and its strong reflections on the Portuguese labor market conditions provided a context of “natural experiment” for assessing health outcomes of individual unemployment status and to develop strategies to minimize their potential mental health effects.

This Delphi panel study contributes toward the designing of effective interventions for mental health promotion among unemployed workers. The participants come from diverse backgrounds, each offering their experience-based perspective on relevant scientific, clinical/intervention, and policy-making areas. The process included a careful selection of experts with focus on their level of understanding of the issue, awareness of need for changes, and knowledge about realistic possibilities for preventive-actions’ implementation.

The most relevant findings are related to which theoretical and psychosocial frameworks are most adequate for this type of intervention, which skills-building are priorities for increasing the chances for future employment and mental health, and which indicators should be considered as control variables and as main outcomes for effectiveness evaluation.

In line with the already existing literature, these Delphi panel results strongly highlighted that the design of the interventions need to consider previous unemployment experiences (and not only the current unemployment situation). In line with this, numerous studies have suggested the positive association between frequency of unemployment and divorce (25, 26), family violence (27, 28), depression (29, 30), self-reported health (31), and suicide (32) especially among males (33). Concerning the skills-building of unemployed people, our findings suggest that it is important that interventions are focused on emotional self-regulation, ability to express one’s own ideas, as well as job search skills. This last issue has been frequently used for interventions among unemployed people, since it is a predictor of job seeking behavior and a catalyst for the increase in well-being (30).

Financial support has an important role during periods of unemployment. This study found that a particular attention should be given to the way individuals perceive financial support at times of unemployment. Other studies showed the mediating role of social benefits in coping with unemployment (34, 35). Considering the maximization of (re)employment opportunities, experts have suggested that interventions should include perception of its impact on employment probability. This agrees with other relevant published research, suggesting that such interventions can result in increased job seeking confidence (self-efficacy), thus increasing the probability of acquiring employment (36, 37). Finally, our results suggested that the intervention should focus on enhancing the ability to cope with the unemployment situation. The relationship between coping skills and well-being has been recognized from previous research in the field of unemployment (38–41).

Regarding the format of the sessions, the expert’s opinions were comparable to other relevant interventions among unemployed. Previous studies have indicated weekly sessions (42, 43) as well as daily sessions (43–45). The duration of the intervention tend to range from 3 h (42) to half day (36, 45, 46). In almost all identified studies, sessions took place in public training units or in employment agencies.

Our findings suggested the importance of two fundamental aspects in tailoring the intervention: mental health literacy improvement (mainly regarding recognition of signs and symptoms of depression and anxiety, and mental health stigma), and interpersonal skills training (e.g., assertive behavior, adequate verbal/non-verbal communication) for increasing the probability of employment. Regarding the assessment of effectiveness, experts expressed the need to assess mental health outcomes and job-searching indicators, as well as perception of utility of the intervention from the participants’ perspective.

Limitations from this Delphi study include the drawbacks associated with the method itself. Main concern relates with its strong dependence on the choice of the experts who are supposed to be true representative and contributors from their respective field. This research attempted to overcome this issue by selecting experts from diverse backgrounds and regions of the country, with extensive knowledge on the problems and potential for intervention in the area. A major strength of this study is the high participation rate of invited experts, from diverse professional backgrounds.

The findings from this study are relevant when translated into the Portuguese context for different reasons: (1) in the last 10 years, Portugal faced higher rates of unemployment when compared with the OECD average, together with one of the most unequal income distribution in Europe (in 2015, the Gini Index for Portugal was 0.34 versus 0.32 for OECD’s average) (2, 4, 47). One of the main reasons for inequality in Portugal is the head of households’ level of education attainment, which ranks Portugal as the country with the third largest share of adults without secondary education (2, 47); (2) one in five Portuguese is affected by at least one mental health disorder (mainly, anxiety and/or mood disorders), with difficult access to mental health care (48, 49); (3) according to the Hofstede’s cultural framework, Portugal is characterized as having a risk-averse culture, with the tendency for short-term orientation and a collectivist nature. These may be some of the contributing factors that position the event of unemployment as a very relevant stress factor affecting the economic and social well-being of both the individual and its nuclear family (50, 51); (4) increase of unemployment rates has been associated with greater incidence of suicide in Portugal (14). Therefore, it is imperative to prevent this possible chain reaction; (4) research suggests that Portuguese (and most particularly, Portuguese men) feel embarrassed to ask for help in this kind of situations because they perceive it as a social failure and loss of standing (52, 53), increasing the risk of silent suffering and mental health aggravation; and (5) public employment services in Portugal are centrally managed and allow the identification of individual’s at the start of their unemployment status (47). This provides the adequate opportunity to intervene on two levels: (a) re-employment potential—increasing the capacities to find a new job (main focus of already existing interventions) and (b) mental health—promoting cognitive, emotional, and behavioral skills to cope adequately with the adversity of the new situation.

Unemployment represents a multidimensional problem and implies multi-sectoral solutions. The same applies to mental health promotion in general and to unemployment-related mental health promotion in particular. The main outcome of this study is that any effective and sustainable intervention needs to be tailored to specific groups of unemployed individuals and needs to be nested in existing inter-sectoral initiatives. Adequate partnerships between different sectors (namely health, social welfare, education) remain the most effective pathway to reduce mental health inequity and to overcome social epidemiology related challenges.

## Ethics Statement

The study was approved by the Centro Académico de Medicina de Lisboa (CAML) and the Comissão Nacional de Proteção de Dados (CNPD). Consent procedure: participants received an online informed consent, together with the questionnaire forms, informing about (i) procedures, aims, methods, benefits, and risks of participation, (ii) voluntary participation, and (iii) freedom to withdraw from the study at any time without consequences, and (iv) the privacy and confidentiality of collected data.

## Author Contributions

OS, EL, and AV designed the study and wrote the protocol with inputs from MH, AD, and SA; and performed data analysis and interpreted the results. OS and AV did the initial survey form, with the review of EL, AD, and SA. EL was responsible for the recruitment of participants and data collection. MP, OS, and EL wrote the first draft of the manuscript. All authors reviewed the article.

## Conflict of Interest Statement

The funder was not involved in the study design and did not contribute to data collection, analysis, and interpretation of data or manuscript writing. The authors declare that the research was conducted in the absence of any commercial or financial relationships that could be construed as a potential conflict of interest. The authors take complete responsibility for the integrity of the data and the accuracy of the data analysis.
