# Supplementary material for: Defining a Brief Intervention for the Promotion of Psychological Well-being among Unemployed Individuals through Expert Consensus
Source: Front Psychiatry. 2018 Feb 7;9:13. doi: 10.3389/fpsyt.2018.00013 (PMC5808332; doi:10.3389/fpsyt.2018.00013)
Supplement: Supplementary file 1 [file table_1.docx]

**Supplementary material**

**Supplementary Table 1. Non-consensual items from the Delphi panel**

| **Items dropped in Round 1** |
| --- |
| **A -** Paradigms of intervention |
| Intervention based on vocational rehabilitation |
| Intervention based on commitment and acceptance therapy |
| Model-based intervention in positive psychology |
| Intervention based on personal development needs |
| Intervention based on social learning and self-efficacy model (Bandura) |
| **B -** Relational/interpersonal strategies |
| Practical exercises-Altruism |
| Practical exercises-functional optimism |
| Practical exercises-Cognitive training |
| Practical exercises-Mindfulness (to negative emotions and recording them in a diary) |
| Intra-group discussion of videos |
| Brainstorming for problem diagnosis and resolution |
| Reflection tasks |
| Compiling individual inter-session registers (i.e. behaviour, emotions) |
| **D –** Contents of the intervention |
| Practical training - job search strategies |
| Practical training - identifying research opportunities through social networks |
| Practical training - asses the (financial and welfare) quality of the job offer and choose between potential options |
| Anticipatory thinking training (how to think like an employer) |
| Cognitive behavioural strategies to reinforce self-efficacy for job search |
| Cognitive behavioural strategies to reinforce self-esteem for job search |
| Prevention of setbacks: identifying and preventing possible barriers to success |
| Training on how to identify and establish sources of emotional and instrumental support |
| Preparations for the workplace: finding social support in the workplace, training on socially acceptable behaviours for the workplace |
| **E –** Contents/skills to be developed/promoted |
| Resilience to adversity |
| Self-stimulation |
| Perceived self-efficacy for job search |
| Perceived self-efficacy at work |
| Cognitive functioning regarding employment |
| Locus of control related to job search strategies |
| Training of coping skills (active and functional) |
| Self-determined motivation for job search |
| **F -** Sociodemographic and professional indicators |
| Ethnicity |
| Nationality |
| Mother language |
| Other fluently spoken languages |
| Criminal record |
| Professional occupation of parents (in case the respondent is a young person looking for a first-job) |
| **H -** Unemployment financial support |
| Frequency of unemployment benefits (i.e. once time only, monthly) |
| Duration of unemployment benefits |
| Based on you initial expectation, how satisfied is you with the received unemployment benefits? |
| Are the unemployment benefits lower, equal or greater compared with the salary from your last job? |
| **I -** Participants satisfaction with the intervention**?** |
| Social skills of the trainer (i.e. enthusiast, sincere etc.) |
| Satisfaction with practical exercises for job search |
| Satisfaction with the group |
| Satisfaction with the working environment |
| **J -** Mental health indicators/outcomes |
| Quality of life |
| Life satisfaction |
| Happiness |
| Optimism |
| Self-esteem |
| Depression |
| Hopelessness |
| Suicidal intentions |
| Risk of suicide |
| Agoraphobia |
| Emotional liability |
| Neuroticism |
| Risk behaviours (i.e. violent, impulsive) |
| Coping strategies |
| Smoking |
| Alcohol consumption |
| Consumption of illicit psychoactive substances |
| Consumption of anxiolytics |
| Consumption of anti-depressives |
| Daytime drowsiness |
| Physical problems |
| **K -** (Re)employment capability |
| Perceived self-efficacy for job search |
| Remaining days until next job |
| Number of times that CV was sent to employers |
| Frequency of contacts with potential employers |
| Number of job interviews attended |
| Attainment of job/internship |
| Type of contract in new job |
| Stability of new job |
| Satisfaction with remuneration in new job |
| Perceived value of new job |
| **L –** Training for burnout prevention and reduction among the staff of the Institute of Employment and Professional Training (IEFP) |
| Identifying and managing bullying/mobbing at work |
| Competence of self-knowledge, empathy and social conscience |
| **Items dropped in Round 2** |
| **A -** Paradigms of intervention |
| Intervention based on analytical models (i.e. group analytic model) |
| Therapy based on metallization (Fonagy) |
| Intervention based on family therapy (dealing with the negative implication of unemployment on the family) |
| Rational-emotional therapy (Albert Ellis) to correct dysfunctional beliefs |
| Mindfulness-based intervention (to promote emotional self-regulation) |
| **B -** Relational/interpersonal strategies |
| Group- tailored intervention strategy |
| **F -** Sociodemographic and professional indicators |
| Housing arrangements |
| Social characteristics (i.e. educational level/occupation of the parents/spouse) |
| Perception of social employment policies |
| Perception of social inequalities at work |
| Self-compassion |
| **I -** Participants satisfaction with the intervention**?** |
| Satisfaction with the technical equipment |
| Satisfaction with location and working conditions |
| Satisfaction with access to the premises |
| **J -** Mental health indicators/outcomes |
| Somatic complaints |
| Changes in consumption (i.e. alcohol, cigarettes) in response to the unemployment situation |
| **L –** Training for burnout prevention and reduction among the staff of the Institute of Employment and Professional Training (IEFP) |
| Training and motivation for participatory citizenship |
| Providing a non-directive space for spontaneous participation |
| Identification of mental health risk symptoms among technicians and how to intervene |
